# On-Chip Electrochemical Sensor Based on 3D Graphene Assembly Decorated Ultrafine RuCu Alloy Nanocatalyst for In Situ Detection of NO in Living Cells

**DOI:** 10.3390/nano15060417

**Published:** 2025-03-08

**Authors:** Haibo Liu, Kaiyuan Yao, Min Hu, Shanting Li, Shengxiong Yang, Anshun Zhao

**Affiliations:** 1 Technology Inspection Center of ShengLi Oil Filed, China Petrochemical Corporation, Dongying 257000, China; liuhaibo330.slyt@sinopec.com; 2 Henan Key Laboratory of Cancer Epigenetics, Cancer Institute, The First Affiliated Hospital, College of Clinical Medicine, Henan University of Science and Technology, Luoyang 471003, China; 230320201168@stu.haust.edu.cn; 3 Key Laboratory of Material Chemistry for Energy Conversion and Storage, Ministry of Education, School of Chemistry and Chemical Engineering, Huazhong University of Science & Technology, Wuhan 430074, Chinam202470398@hust.edu.cn (S.L.); ysx2883577@126.com (S.Y.)

**Keywords:** cell chip, electrochemical sensor, 3D graphene assembly, ultrafine alloy nanocatalyst, in situ NO detection

## Abstract

In this work, we developed 3D ionic liquid (IL) functionalized graphene assemblies (GAs) decorated by ultrafine RuCu alloy nanoparticles (RuCu-ANPs) via a one-step synthesis process, and integrated it into a microfluidic sensor chip for in situ electrochemical detection of NO released from living cells. Our findings have demonstrated that RuCu-ANPs on 3D IL-GA exhibit high density, uniform distribution, lattice-shaped arrangement of atoms, and extremely ultrafine size, and possess high electrocatalytic activity to NO oxidation on the electrode. Meanwhile, the 3D IL-GA with hierarchical porous structures can facilitate the efficient electron/mass transfer at the electrode/electrolyte interface and the cell culture. Moreover, the graft of IL molecules on GA endows it with high hydrophilicity for facile and well-controllable printing on the electrode. Consequently, the resultant electrochemical microfluidic sensor demonstrated excellent sensing performances including fast response time, high sensitivity, good anti-interference ability, high reproducibility, long-term stability, as well as good biocompatibility, which can be used as an on-chip sensing system for cell culture and real-time in situ electrochemical detection of NO released from living cells with accurate and stable characteristics in physiological conditions.

## 1. Introduction

Endogenous nitric oxide (NO), an endothelial derived relaxation factor, plays a vital role in various pathological and physiological processes, including immune response [1], wound healing [2], vasodilation [3], cancer biology [4], gastrointestinal physiology [5], and neurotransmission [6]. Therefore, the monitoring of NO in cells and organs can provide important insight into understanding different pathologies caused by abnormal NO levels, such as sepsis [7], Parkinson’s [8], atherosclerosis [9], ischemia-reperfusion injury [10], and cancer progression [11]. However, the in situ real-time tracking of NO in complex physiological and biological environments is greatly challenging due to its small molecular size, short half-life of 6~10 s [12], low concentration down to several nanomoles [13], high diffusion constant up to 3300 μm s^−2^ [14], and high chemical activity and interference by other chemicals [15]. Therefore, it is highly desirable to develop sensitive and robust analytical methods and high-performance devices for NO detection.

Among various analysis methods, electroanalysis possesses several superior properties such as high sensitivity, low detection limits, fast response, as well as cost-effectiveness, portable instruments, and easy implementation [16,17,18]. Currently, various smart electrochemical NO sensors based on highly active electrode materials have been developed and exhibited good performances in real-time and in vivo NO monitoring [15,19,20,21,22]. Significantly, microfluidic sensor chips offer several advantages including miniaturization, ease of integration, low sample consumption, and rapid analysis [23,24]. Several groups, including ours, have demonstrated that the integration of microelectrodes into electrochemical microfluidic chips can endow the resultant electrodes with high spatial and temporal resolution, which provides a highly efficient platform for in situ sensitive detection of various electrochemical active biomarkers such as NO, H_2_O_2_, H_2_S, etc., from living cells and various human samples [25,26,27,28,29,30]. However, in order to improve the overall sensing performances in NO detection and enable the in situ continuous NO monitoring, we still need to explore new types of electrode materials characterized by exceptional electrocatalytic activity and selectivity toward the oxidation reaction of NO in a complex biological environment, and miniaturized electrochemical sensor devices for cell culture and simultaneous detection of NO released from living cells.

Recently, various 2D nanomaterials, e.g., graphene, MXene, hexagonal boron nitride (h-BN), borophene, phosphorene, transition-metal dichalcogenide (TMD), and 2D metal organic framework (2D-MOF) [31], have sought great attention due to their astonishing features such as layered morphology, high surface-to-volume ratio, anisotropy, magnificent electrical properties, high thermal and chemical stability, as well as tunable interlayer spacing and multi-functionality [32,33], which is of great significance in catalytic, biosensing, and biomedical systems [34,35,36]. Specifically, 2D graphene oxide and MXene nanosheets, with abundant surface groups and π-electron structure, can be self-assembled or co-assembled into various flexible 3D macroscopic architectures [37,38], with complex structures including shape, size, and functionality [39,40,41]. The as-obtained 3D assembles inherit the intrinsic physicochemical properties and good biocompatibility of 2D graphene and MXene, and exhibit unique characteristics of light weight and extraordinary mechanical strength [42,43]. These outstanding properties make 3D-assembled materials a wonderland for green energy conversion [44], disease therapy [45], biosensing [46,47], and wearable devices [48].

**Figure 1:**
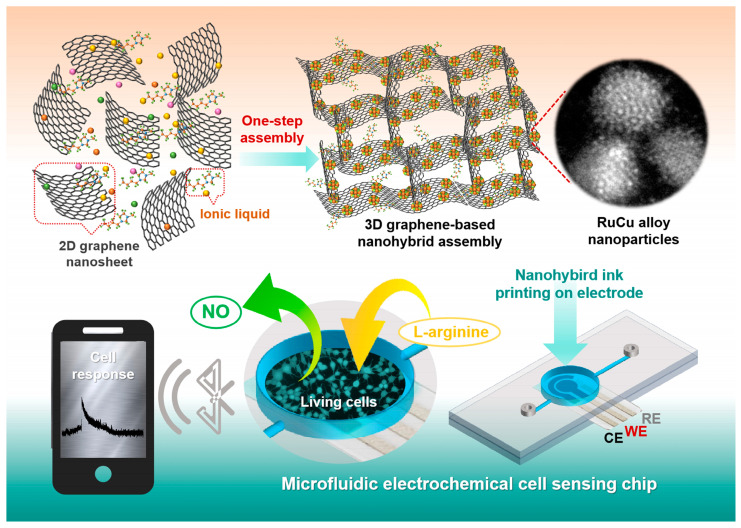
Schematic depiction for the synthesis of RuCu-ANPs/IL-GA nanohybrid electrode material and the fabrication of a microfluidic electrochemical cell sensing chip.

In this work, we developed an electrochemical microfluidic sensor chip based on 3D graphene assembly (GA) decorated by ultrafine RuCu alloy nanoparticles (RuCu-ANPs). As shown in Figure 1, the 3D graphene-based nanohybrid was synthesized by a facile one-step process via assembling graphene oxide nanosheets (GONs) in the presence of imidazoles ionic liquid (IL, i.e., 1-butyl-3-methylimidazolium tetrafluoroborate, [BMIM]BF_4_) and different metal precursors. By cation–π interaction between π-electron structure on GONs and the imidazolium anion of [BMIM]BF_4_, the IL molecules will graft on the surface of GONs, which not only prevents the aggregation of GONs in the self-assembly process to form 3D architectures, but also provides a large amount of active sites for the nuclear growth of RuCu-ANPs on it, as the anions of IL and numerous negative functional groups on GO nanosheets can interact with metal cations by electrostatic interaction. Furthermore, the graft of [BMIM]BF_4_ molecules on graphene can endow it with high hydrophilicity, which can be synthesized into aqueous ink and printed on any electrode substrate. Our findings demonstrate that high-density RuCu-ANPs on 3D IL grafted GA (IL-GA) exhibit a lattice-shaped arrangement of atoms that have an extremely ultrafine size of ~2 nm, and possess highly electrocatalytic activity to NO oxidation on the electrode. Meanwhile, the 3D IL-GA with a hierarchical porous structure can facilitate the efficient electron/mass transfer at the electrode/electrolyte interface and the cell culture as well. Therefore, the resultant electrochemical microfluidic sensor demonstrated excellent sensing performances including fast response time, high sensitivity, good anti-interference ability, high reproducibility, long-term stability, as well as good biocompatibility. The practical application of the proposed sensor has been explored by using it as a cell chip for cell culture and in situ detection of NO released from living cells, which could contribute to the accurate and stable tracking of NO levels in complex physiological conditions providing essential diagnostic and therapeutic information.

## 2. Experimental

### 2.1. Characterization

Spherical aberration-corrected scanning transmission electron microscopy (AC-STEM, FEI, Themis Z, Thermo Fisher Scientific, Waltham, MA, USA), high-resolution transmission electron microscopy (HR-TEM, FEI Tecnai G2 F30, FEI, Hillsboro, OR, USA), scanning electron microscope (SEM, Zeiss, Oberkochen, Germany) and energy-dispersive X-ray spectrometer (EDS) were employed for the characterization of the nanostructure and composition. X-ray diffraction (XRD) pattern was collected on a Rigaku SmartLab SE (Rigaku, Tokyo, Japan) with scan range from 10° to 80° at 5° min^−1^. X-ray photoelectron spectroscopy (XPS) measurement was tested using a Perkin-Elmer model PHI 5600 XPS system (PerkinElmer, Waltham, MA, USA), with all peaks corrected by default for the C 1s line at 284.6 eV. Cyclic voltammetric (CV) and chronoamperometric experiments were performed with the miniature electrochemical workstation (CS 100E) using a three-electrode system.

### 2.2. Synthesis of RuCu-ANPs/IL-GA

GO was synthesized from natural graphite by the modified Hummers method [49]. Then, 26 mg (0.1 mmol) Cu(NO_3_)_2_∙3H_2_O and 10 mg (0.05 mmol) RuCl_3_·3H_2_O were dissolved in 5 mL homogeneous GO aqueous dispersion (5 mg mL^−1^) with 10% imidazoles IL (i.e., 1-butyl-3-methylimidazolium tetrafluoroborate, [BMIM]BF_4_). Subsequently, 50 g diethylene glycol was added into the above solution under sonication for about 1 h. Then, the mixture was sealed in a 50 mL Teflon-lined autoclave and maintained at 180 °C for 2 h. After the autoclave was naturally cooled to room temperature, a black gel-like 3D graphene cylinder (i.e., graphene hydrogel) was obtained. During these processes, RuCu-ANPs were also grown on 3D graphene architecture. The RuCu-ANP-decorated graphene hydrogel was finally freeze-dried overnight for the following experiment, and the as-obtained graphene-based nanohybrid aerogel was re-dispersed in water (10 mg mL^−1^) to form aqueous 3D IL functionalized GA decorated by ultrafine RuCu-ANPs (RuCu-ANPs/IL-GA) ink. For comparison, Ru-NPs/IL-GA and Cu-NPs/IL-GA were prepared under the same procedure with the addition of different metal precursors, and pristine IL-GA was also prepared without the addition of any metal precursors.

### 2.3. Preparation of Three-Electrode System

The flexible three-electrode system is a flexible screen-printed electrode (FSPE) purchased from Poten Technology Co., Ltd. (Weihai, China), which was fabricated on a polyimide (PI) substrate using photolithography and electroplating techniques (Supplementary Materials, Figure S1). The working electrode (WE) is printed activated carbon material and the reference electrode (RE) is printed Ag/AgCl. When in use, the different 3D graphene-based nanohybrid ink is modified on WE. In detail, 10 mg RuCu-ANPs/IL-GA was dispersed in 750 μL ethanol and 250 μL deionized water solvent containing 5 μL Nafion (5 wt%). Subsequently, 10 μL as-prepared mixture ink was drop-cast onto WE and air-dried at ambient surrounding for one night. All potentials were measured vs. Ag/AgCl electrode.

### 2.4. Fabrication of Microfluidic Electrochemical Sensing Chip

The flexible microfluidic electrochemical sensing chip was fabricated according to our previous work [27]. The chip was designed using AutoCAD 2020 software, and then fabricated by lithography, mask preparation, etching, PDMS curing, and bonding processes. The entire chip is 55 mm in length and 25 mm in width, and consists of four layers. The liquid flow channel is 0.5 mm wide and 0.5 mm high, and the detection chamber is designed to fit the area of WE, where the living cells will grow over the 3D IL-GA of WE through the flow channel. The released NO from cells will be detected by WE. The FSPE was then connected to the handheld electrochemical workstation for signal analysis. The data display and wireless communication are used by an Android phone via a Bluetooth system.

### 2.5. Cell Culture in Sensing Chip for Real-Time Monitoring

To construct a cell sensor chip, the human breast cancer cell lines (MCF-7) purchased from the American Type Culture Collection (ATCC, Manassas, VA, USA) were cultured in Dulbecco’s modified Eagle’s media (Gibco, Waltham, MA, USA), supplemented with 10% fetal bovine serum (FBS, ScienCell, Carlsbad, CA, USA), 100 units mL^−1^ penicillin, and 100 µg mL^−1^ streptomycin. The cell culturing was conducted in a humidified atmosphere with 5% CO_2_ at 37 °C, with subculturing performed every 3 days. The living cells were harvested via centrifugation when they reached ~80% confluence (5 × 10^6^ cells mL^−1^) [29]. The cell suspension was pumped into the sensor chip through the microfluidic channel and at a flow rate of 20 µL min^−1^, and then, the microfluidic chip inoculated with cells was placed in the incubator for 6 h to allow the cells to grow in situ on the WE surface, which was used for real-time and continuous detection of NO released from MCF-7 cells.

## 3. Results and Discussion

### 3.1. Morphological and Structural Characterization

**Figure 2:**
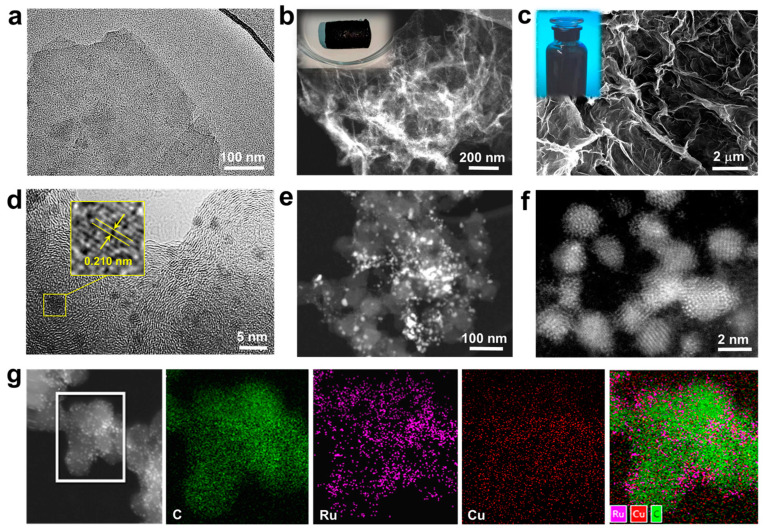
(**a**) TEM image of GON; (**b**) dark-field TEM image of IL-GA, inset of (**b**) is the photograph of IL-GA hydrogel cylinder; (**c**) SEM images of IL-GA, inset of (**c**) is the photograph of IL-GA dispersed in aqueous solution to form a homogenous ink; (**d**) TEM image and (**e**) dark-field TEM image of RuCu-ANPs/IL-GA. Inset of (**d**) is the HR-TEM image of RuCu-ANP on IL-GA; (**f**) AC-STEM image of RuCu-ANPs on IL-GA. (**g**) EDX elemental mapping images of C, Ru, and Cu and elements over IL-GA.

The morphology and composition of the graphene-based nanohybrid materials were first characterized by TEM. Figure 2a demonstrates that GO synthesized from natural graphite exhibited typical nanosheet morphology. Upon the hydrothermal reduction at 180 °C for 2 h, the GONs are self-assembled into a 3D hydrogel cylinder in the presence of imidazoles IL [BMIM]BF_4_ and different metal precursors (Figure 2b inset). By cation–π interaction between IL and GONs, the IL molecules will graft on the surface of GONs, which can effectively prevent the aggregation of GONs in the self-assembly process. As shown in the dark-field TEM image of Figure 2b, the 3D IL-GA is composed of nanosheets with several micro-/nano-pores through the assembly. Due to the incorporation of hydrophilic [BMIM]BF_4_, the as-obtained IL-GA-based materials can be well-dispersed in water to form a homogeneous ink (Figure 2c inset), and the micro-/nano-porous structure is still visible from the SEM image of the IL-GA ink (Figure 2c).

Figure 2d shows the TEM image of RuCu-ANPs/IL-GA. The porous and network structure of 3D IL-GA enables highly concentrated surface active sites, which can strongly absorb metal cation precursor, providing lots of active sites for the nucleation and growth of RuCu-ANPs. By in situ reduction of the metal precursor, high-density RuCu-ANPs, with an ultrafine size of ~2 nm, are uniformly dispersed on the surface of graphene nanosheets. The HR-TEM image shows that the lattice fringes of 0.210 nm can be attributed to the (002) plane of Ru [50] (Figure 2d inset). Evidently, the incorporation of Cu atoms should account for the smaller lattice spacing of RuCu relative to bulk Ru (0.214 nm) [51]. The dark-field TEM image of Figure 2e shows that the 3D IL-GAs are entirely decorated by high-density RuCu-ANPs. Furthermore, the nanostructures of RuCu-ANPs have been investigated by AC-STEM. As shown in Figure 2f and Figure S2, the ultrafine RuCu-ANPs are assembled by several Ru and Cu atoms, which possess a nanocrystallite size, lattice structure and strains, and interface defect states, and are anticipated to possess extremely high electrocatalytic activity. Furthermore, EDX elemental mapping images indicate the uniform distribution of C, Ru, and Cu elements over IL-GA (Figure 2g). The mass ratio of Ru and Cu in RuCu-ANPs/IL-GA are determined to be 0.54% and 0.33%, respectively (Figure S3).

**Figure 3:**
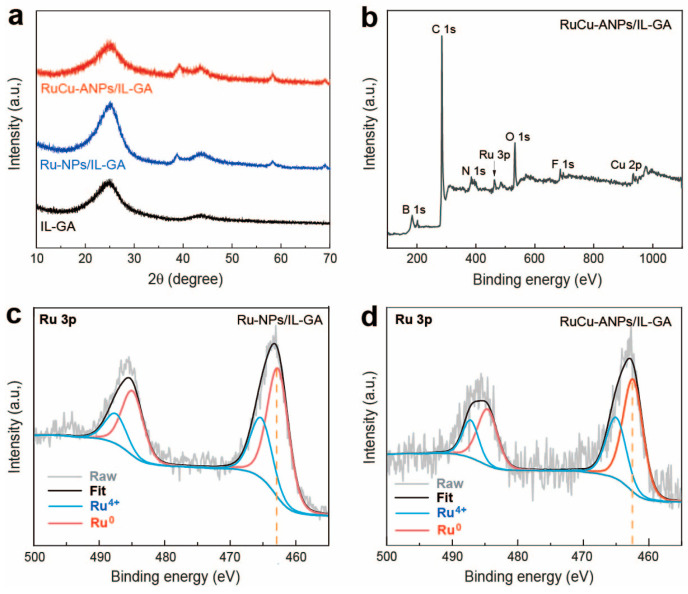
(**a**) XRD pattern of RuCu-ANPs/IL-GA, Ru-NPs/IL-GA, and IL-GA. (**b**) XPS survey spectrum of RuCu-ANPs/IL-GA. High-resolution XPS spectra of Ru 3p regions in (**c**) Ru-NPs/IL-GA and (**d**) RuCu-ANPs/IL-GA.

The nanostructure and chemical composition of different samples have been analyzed by XRD and XPS. As shown in Figure 3a, the XRD patterns of both RuCu-ANPs/IL-GA and Ru-ANPs/IL-GA show two diffraction peaks at 38.5° and 58.5°, indicating the presence of Ru [52]. In addition, for RuCu-ANPs/IL-GA, Ru-ANPs/IL-GA, and IL-GA samples, the broad reflection at 25° corresponds to the carbon species of graphene. Figure 3b shows the XPS survey spectrum of RuCu-ANPs/IL-GA, which demonstrates the existence of C, O, N, B, F, Ru, and Cu in the nanohybrid material, and the atomic ratio of Ru and Cu in RuCu-ANPs/IL-GA are determined to be 0.8% and 1.2%, respectively. The high-resolution Ru 3p XPS spectra of both Ru-NPs/IL-GA and RuCu-ANPs/IL-GA are shown in Figure 3c,d, respectively. It can be observed that the Ru 3p_3/2_ spectrum in Ru-NPs can be deconvoluted into two characteristic peaks centered at 462.7 eV and 465.3 eV, corresponding to Ru^0^ and Ru^4+^, respectively. Significantly, the peaks of Ru 3p_3/2_ in RuCu-ANPs shift towards lower binding energy by 0.3 eV compared to that of Ru-NPs. The chemical shifts of Ru 3p binding energies indicate that Cu atoms are incorporated into Ru-NPs lattices with a certain amount of the electrons transferring from Cu atoms to Ru atoms [53,54]. This confirms the successful synthesis of RuCu alloy species. In addition, as shown in Figure S4, the Cu 2p_3/2_ spectrum in RuCu-ANPs can be deconvoluted into two characteristic peaks centered at 933.1 eV and 935.3 eV, corresponding to Cu^0^ and Cu^2+^, respectively; these observations are consistent with the previous reports [54].

### 3.2. Electrochemical Catalytic and Sensing Performances

**Figure 4:**
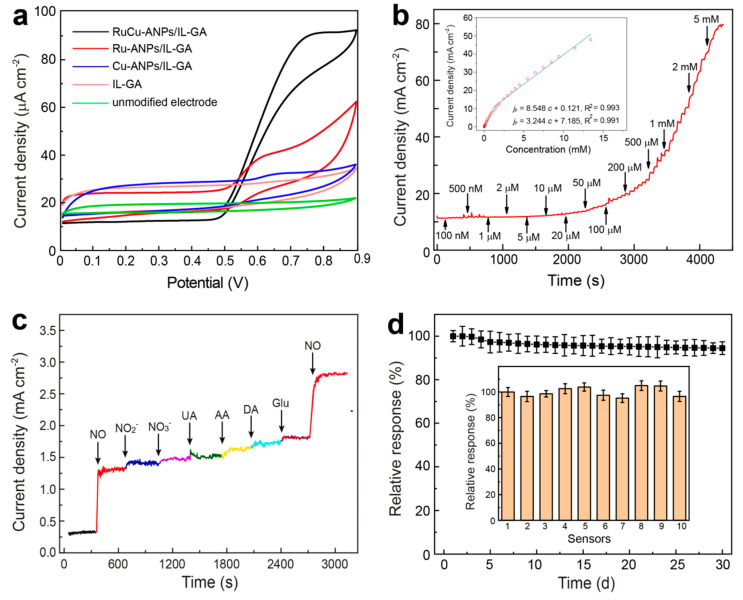
(**a**) CV curves of different electrodes in 0.1 M PBS (PH 7.0) containing 0.5 mM NO, scan rate: 50 mV s^−1^. (**b**) Amperometric current response of RuCu-ANPs/IL-GA-based sensor to successive addition of NO in PBS at 0.74 V (vs. Ag/AgCl); inset of (**b**) is the linear dependence of the amperometric current response vs. NO concentration. (**c**) Amperometric curve to 3.0 mM NO_3_^−^ and NO_2_^−^, and 1.0 mM UA, AA, DA, Glu and 0.3 mM NO. (**d**) Relative current responses of 0.3 mM NO on the same sensor after being stored for different days; inset of (**d**) is the relative current responses of ten sensors towards 0.3 mM NO. Error bars represent the standard deviation from six parallel tests.

The electrochemical catalytic activity of different modified electrodes towards NO has been investigated by cyclic voltammetry (CV) measurements. In the electrochemical tests, a PBS solution (0.1 M, pH 7.4) containing different concentrations of NO was used as the electrolyte. The optimized pH value is consistent with that in physiological conditions. Figure 4a shows the CV responses of the RuCu-ANPs/IL-GA-modified electrode in 0.1 M PBS (pH 7.4) containing 0.5 mM NO, with Ru-NPs/IL-GA-, Cu-NPs/IL-GA-, and IL-GA-modified electrodes and unmodified electrode as the control. For the unmodified electrode, there are no electrochemical responses, showing its poor electroactivity toward NO oxidation. While for the IL-GA-modified electrode, a slight oxidation peak appears due to the quite low electrocatalytic activity to NO in the absence of metal nanocatalyst. For the Ru-NPs/IL-GA and Cu-NPs/IL-GA electrodes, it is evident that Ru-NPs/IL-GA had enhanced electrocatalytic activity evidenced by its more negative peak potential, as well as a higher peak current density than that of Cu-ANPs/IL-GA. Furthermore, in the CV curve of the RuCu-ANPs/IL-GA-modified electrode, a distinct oxidation peak can be observed at an anodic potential of 0.74 V. Dramatically, RuCu-ANPs/IL-GA results in dramatically increased anodic peak current density in comparison to that of Ru-NPs/IL-GA and Cu-NPs/IL-GA.

These results collectively demonstrate that the remarkably improved electrocatalytic activity of the RuCu-ANPs/IL-GA-modified electrode is due to the synergistic contribution of RuCu-ANPs electrocatalyst and IL-GA electrode substrate. The high-density and ultrafine RuCu-ANPs on 3D micro-/nano-porous graphene assembly furnish an extensive surface area, abundant active sites and efficient mass/electron transfer channels. More importantly, the exceptional catalytic activity of RuCu-ANP systems stems from two primary factors, i.e., the “bifunctional effect” relates to geometric alterations in alloy systems vs. monometallic ones, and the “ligand or electronic effect” signifies changes in the electronic characteristics of pure metals upon the addition of a second metal component [15,55]. These effectively promote the electro-oxidation reaction of NO on the RuCu-ANPs/IL-GA electrode. During NO electrochemical oxidation, NO loses an electron to form NO^+^ (nitrosonium ion) on the surface of the RuCu-ANPs/IL-GA electrode, followed by a subsequent conversion of NO^+^ to NO_2_^−^ by the following reaction [19,56]:

NO − e^−^ → NO^+^ (1)

NO^+^ + OH^−^ → HNO_2_ (2)

The amperometric measurements were performed upon successive addition of NO in PBS solution (0.1 M, pH 7.4) at the optimized applied potential is 0.74 V. Figure 4b shows the amperometric current response of the RuCu-ANPs/IL-GA electrode upon successive addition of NO in PBS (0.1 M, pH 7.4). When a certain concentration of NO solution was added to stirred PBS, the RuCu-ANPs/IL-GA electrode responded rapidly and a 95% steady state current response was obtained within 5 s. The Figure 4b inset shows the calibration curve of the amperometric responses of the RuCu-ANPs/IL-GA electrode to NO concentration. The linear ranges of the proposed electrode for the estimation of NO are found to be in two ranges of 100 nM~960 μM and 960 μM~13.4 mM, with a high sensitivity of 8.548 and 3.244 mA cm^−2^ mM^−1^, respectively. At low concentrations, the electrochemical reaction of NO on electrode is mainly controlled by adsorption, while it is mainly controlled by diffusion at high concentrations [57]. The limit of detection (LOD) is as low as 20 nM, which is calculated from the calibration curve by 3*S*_b_*m*^−1^ where *S*_b_ is the standard deviation of the intercept of the calibration curve and *m* is the slope of the calibration curve [19].

### 3.3. The Anti-Interference Ability

The anti-interference ability is very important for electrochemical sensing of NO in real biological samples. Consequently, the proposed RuCu-ANPs/IL-GA-based sensor has been tested for detecting NO in the presence of potentially coexisting electroactive biothiols commonly found in physiological samples, such as nitrate (NO_3_^−^), nitrite (NO_2_^−^), dopamine (DA), uric acid (UA), ascorbic acid (AA), and glucose (Glu). Figure 4c depicts the amperometric responses of the RuCu-ANPs/IL-GA-based sensor upon successive additions of a certain concentration of NO, NO_3_^−^, NO_2_^−^, UA, AA, DA, Glu, and NO at regular intervals in 0.1 M PBS. It can be observed that upon adding 0.3 mM NO in the solution, the amperometric current responses corresponding to NO oxidation increase at an applied potential of 0.74 V, and change less than 5.0% upon the addition of 3.0 mM NO_3_^−^ and NO_2_^−^, and 1.0 mM UA, AA, DA, and Glu. This excellent anti-interference performance can be attributed to the high electrocatalytic activity and selectivity of bimetallic RuCu-ANPs to NO oxidation on the electrode.

### 3.4. Reproducibility and Stability

The reproducibility and stability of RuCu-ANPs/IL-GA-based sensors for detection of NO are further evaluated by amperometric measurement. Ten independent sensors fabricated by the same procedures revealed relative responses from 96.5% to 105.0%, with a relative standard deviation (RSD) of less than 5.0% in response to 0.3 mM NO, indicating high reproducibility of the sensor. Subsequently, ten independent measurements using one sensor show an RSD of less than 4.0%. Moreover, the stability measurement demonstrates that the RuCu-ANPs/IL-GA-based sensor retains 94.5% of its initial sensitivity after 30 d. These indicate the robust stability of the proposed sensor for repeated and long-term NO detection.

### 3.5. On-Chip Detection of NO Released from Living Cells

**Figure 5:**
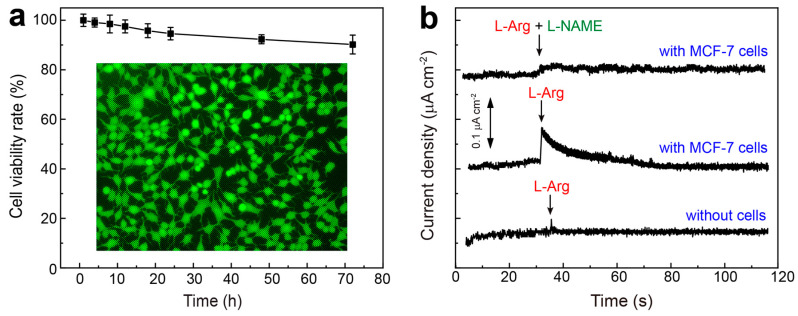
(**a**) Quantitative cell viability results of CCK-8 assay for MCF-7 cells incubated with RuCu-ANPs/IL-GA electrode from 0 to 72 h; inset of (**a**) is the dark-field fluorescent images of MCF-7 cells after the calcein-AM/PI assay to stain the viable cells green by calcein-AM. (**b**) Amperometric current responses of the electrochemical sensor to the addition of 5.0 mM L-Arg and L-NAME into the culture medium in the chip containing MCF-7 cells (5 × 10^6^ cells mL^−1^) by microfluidic channels.

It is well-known that the real-time in situ detection of NO released from living cells provides an important insight into the study of the correlation of NO generation with neuronal signaling and inflammatory responses. In this work, the living cells (i.e., MCF-7) were seeded within the chip, with the cell culture medium pumping through the upper channel into the chip. Upon reaching ~80% confluence (5 × 10^6^ cells mL^−1^), the sensing chip was operated as the detection unit for continuous monitoring. Before electrochemical measurement, a quantitative cytotoxicity study of the proposed electrode to the cells was conducted using the CCK-8 assay. As shown in Figure 5a, the living cells maintain viability rates of 99.1%, 98.5%, 97.5%, 95.8%, 94.6%, 92.3%, and 90.2%, after incubation with RuCu-ANPs/IL-GA-based electrode for 4, 8, 12, 18, 24, 48, and 72 h, respectively. Furthermore, a fluorescence double-staining assay (calcein acetoxymethyl ester/propidium iodide, Calcein-AM/PI) was used to evaluate the cell viability when MCF-7 cells were incubated with RuCu-ANPs/IL-GA for 72 h. It can be observed that these cells are very healthy and emit strong green fluorescence (Figure 5a inset), and the red fluorescence from the dead cells cannot be observed (Figure S5). These indicate good biocompatibility of RuCu-ANPs/IL-GA toward the living cells, which enables it to act as high-performance electrode material in cell chip for real-time in situ monitoring.

In virtue of the excellent sensing performances as well as high stability and good biocompatibility, the RuCu-ANPs/IL-GA-based sensor (Figure S6) has been used in electrochemical morning NO released from MCF-7 cells. It is known that within organisms, living cells can generate NO by the uptake of extracellular L-arginine (L-Arg) under the catalysis of the nitric oxide synthase (NOS). Meanwhile, Nω-nitro-L-arginine methyl ester (L-NAME), a nonspecific inhibitor of NO, can be used to inhibit NO production [58]. Subsequently, the real-time in situ tracking of NO release from MCF-7 cells was performed by adding NOS stimulator L-Arg and inhibitor L-NAME to modulate the concentration of NO. As shown in Figure 5b, upon the addition of 5 mM L-Arg into the culture medium in the chip containing MCF-7 cells (5 × 10^6^ cells mL^−1^) by microfluidic channels, a surge in the amperometric response current is observed, which gradually returns to baseline ~20 s after stimulation. For comparison, no amperometric response is recorded when 5 mM L-Arg is added to the microfluidic chip without cells. This indicates that the rapid increased amperometric current response originates from a certain amount of NO released from living cells. To further support this result, L-Arg and L-NAME have been simultaneously added into the cell culture medium containing MCF-7 cells. Dramatically, there are no chances in the current response, which was attributed to the inhibitory effect of L-NAME on NO release. Importantly, these results are consistent with other reported cells, such as cardiac cells and MCF-7 cells [59], HeLa cells [60], HepG2 and RAW 264.7 cells [61], HUVECs or organ [56,62] and chondrocytes and organ from cartilage of SD rats [19]. Therefore, the proposed on-chip cell sensor can successfully capture NO concentration by real-time and continuous electrochemical monitoring, which can offer a new strategy for the dynamic monitoring of living cells’ state.

## 4. Conclusions

In summary, we developed an electrochemical microfluidic sensor chip based on 3D IL-GA decorated by ultrafine RuCu-ANPs, and explored its practical application as an on-chip sensing system for cell culture and real-time in situ electrochemical detection of NO released from living cells. Our strategy features several advantages: (1) 3D IL-GA assembled from GONs and ILs possesses hierarchical porous structure to facilitate the efficient electron/mass transfer and the cell culture as well, and provides high surface area and abundant active sites for the nuclear and growth of RuCu-ANPs on it; (2) High-density RuCu-ANPs on 3D IL-GA exhibit a lattice-shaped arrangement of atoms and an extremely ultrafine size of ~2 nm, as well as the synergistic effect of bimetallic active sites, which possess highly electrocatalytic activity to NO oxidation on electrode, and improve the sensing performances of the nanohybrid electrode; (3) The innovative integration of nanocatalysts, micro-/nano-processing technology, microfluidic chips, and smartphones can be used as cell chips for real-time in situ electrochemical detection of NO released from MCF-7 cells, with accurate and stable characteristics in physiological conditions. We envision that the engineered optimization of the nanohybrid electrode and innovative design of the integrated sensor chip will facilitate the highly accurate detection of various biologically significant species within cells, tissues, and organs, which will promote real-time clinical research for health monitoring and diagnostic purposes.

## Data Availability

The data presented in this study are available on request from the corresponding author.

## Supplementary Materials

The following supporting information can be downloaded at: https://www.mdpi.com/article/10.3390/nano15060417/s1, Figure S1. Photos of (a) flexible screen printed electrode and (b,c) integrated microfluidic chip. Figure S2. AC-STEM image of RuCu-ANPs on IL-GA. Figure S3. Elemental analysis of RuCu-ANPs on IL-GA. Figure S4. High-resolution XPS spectra of Cu 2p regions in RuCu-ANPs/IL-GA. Figure S5. Amperometric current response of RuCu-ANPs/IL-GA based sensor to successive addition of low concentration (100 nM–10 μM) and medium concentration (20 μM–200 μM) NO in PBS at 0.74 V (vs. Ag/AgCl). Figure S6. Dark-field fluorescent image of MCF-7 cells after the calcein-AM/PI assay to stain the viable cells green by calcein-AM and dead cells red by PI. The living cells were incubated with RuCu-ANPs/IL-GA for 72 h.
