# Supplementary material for: On-Chip Electrochemical Sensor Based on 3D Graphene Assembly Decorated Ultrafine RuCu Alloy Nanocatalyst for In Situ Detection of NO in Living Cells
Source: Nanomaterials (Basel). 2025 Mar 8;15(6):417. doi: 10.3390/nano15060417 (PMC11946219; doi:10.3390/nano15060417)
Supplement: Supplementary file 1 [file nanomaterials-15-00417-s001.zip › nanomaterials-3486856-supplementary.pdf]

Supplementary Materials

On-chip electrochemical sensor based on 3D graphene assembly decorated ultrafine RuCu alloy nanocatalyst for in situ detection of NO in living cells

Haibo Liu[1], Kaiyuan Yao[2], Min Hu[3], Shanting Li[3], Shengxiong Yang[3], Anshun Zhao[2,*]

1 Technology Inspection Center of ShengLi Oil Filed, China Petrochemical Corporation, Dongying 257000, China

2 Henan Key Laboratory of Cancer Epigenetics, Cancer Institute, The First Affiliated Hospital, College of Clinical Medicine, Henan University of Science and Technology, Luoyang 471003, China

3 Key Laboratory of Material Chemistry for Energy Conversion and Storage, Ministry of Education, School of Chemistry and Chemical Engineering, Huazhong University of Science & Technology, Wuhan 430074, China;

*Correspondence: anshunzhao@haust.edu.cn

1. Experimental section

1.1. Materials and reagents

Expanded graphite powder (Qingdao Henglide Graphite Co., Ltd.). Concentrated sulfuric acid (H_2SO_4, 98%), potassium permanganate ($KMnO_4$), hydrogen peroxide (H_2O_2, 30%), hydrochloric acid (HCl, 37%), copper nitrate hydrate ($Cu(NO_3)_2 \cdot 3H_2O$,ACS reagent, 98%), ruthenium trichloride trihydrate ($RuCl_3 \cdot 3H_2O$), nitrite sodium ($NaNO_2$, ACS reagent, ≥97%) potassium hydroxide (KOH, ACS reagent, ≥85%), diethylene glycol were purchased from Aladdin Chemistry Co., Ltd (China). 1-butyl-3-methylimidazolium hexafluorophosphate ([BMIM][BF4]) was obtained from Shanghai Chengjie Chemical Co., Ltd. Deionized water (resistivity: 18.25 Ω cm-1) was prepared by using an RU water purification system (RiOs16, Millipore). All the reagents were used without any further purification.

1.2 Preparation of NO in solution

The saturated NO in PBS solution was prepared according to the reported procedure [1,2]. Before the preparation of NO saturated solution, all apparatus were degassed with nitrogen for 30 min to exclude oxygen. NO gas was generated by slowly dropping 2 M H_2SO_4 into a glass flask containing saturated $NaNO_2$ solution. Then, NO gases were sequentially passed through saturated KOH solution to remove oxygen and other nitrogen oxides. A saturated NO solution (1.8 mM, 20 °C) was prepared by bubbling the generated NO gas into PBS. The saturated NO solution was then diluted with PBS to obtain different NO concentrations for a NO sensor. The NO solution must be prepared and used immediately to ensure reliable NO concentration.

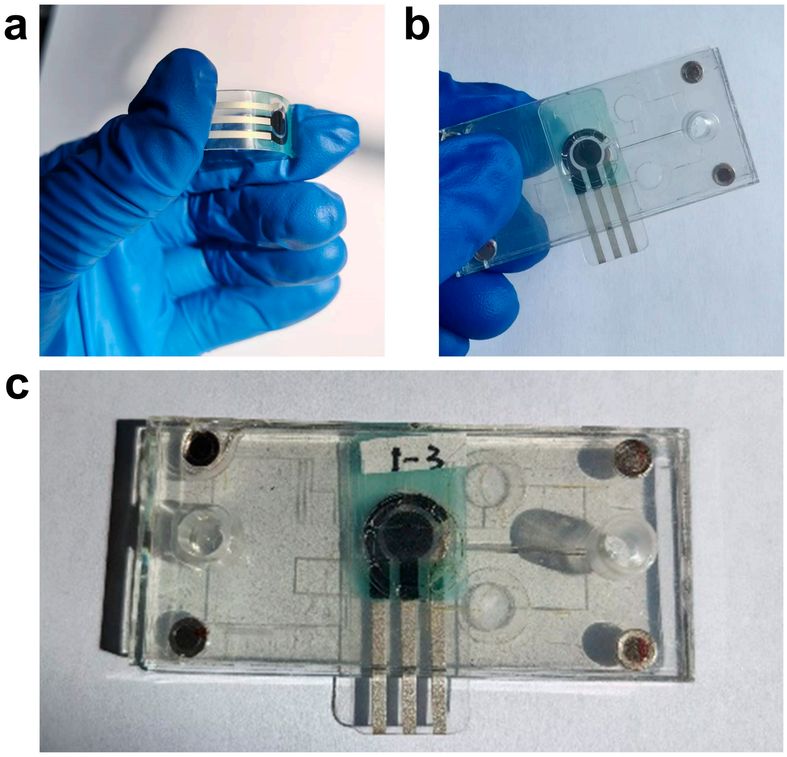

Figure S1. Photos of (a) flexible screen printed electrode and (b, c) integrated microfluidic chip.

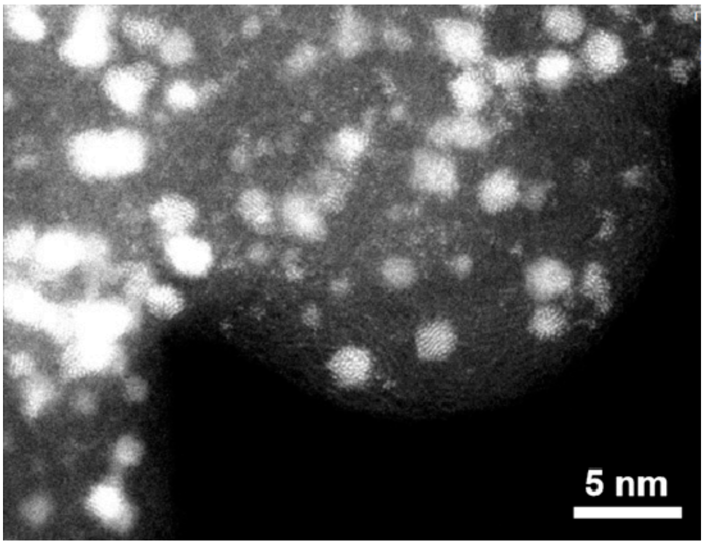

Figure S2. AC-STEM image of RuCu-ANPs on IL-GA.

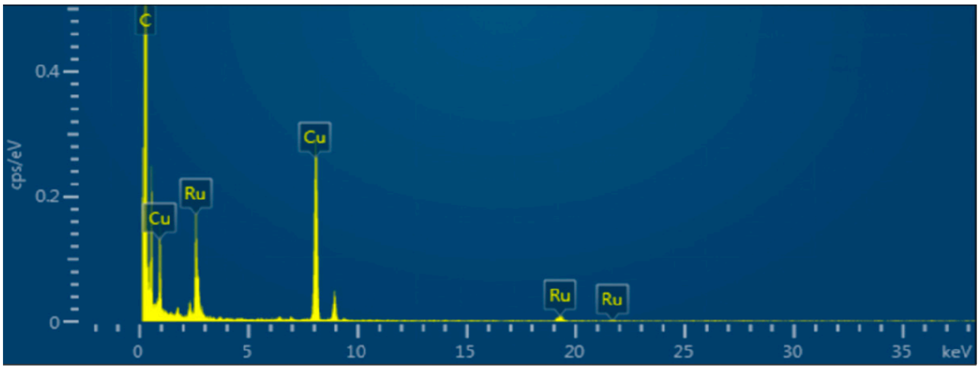

Element	Wt%	At%
C	94.04	99.13
Cu	1.64	0.33
Ru	4.32	0.54

Figure S3. Elemental analysis of RuCu-ANPs on IL-GA.

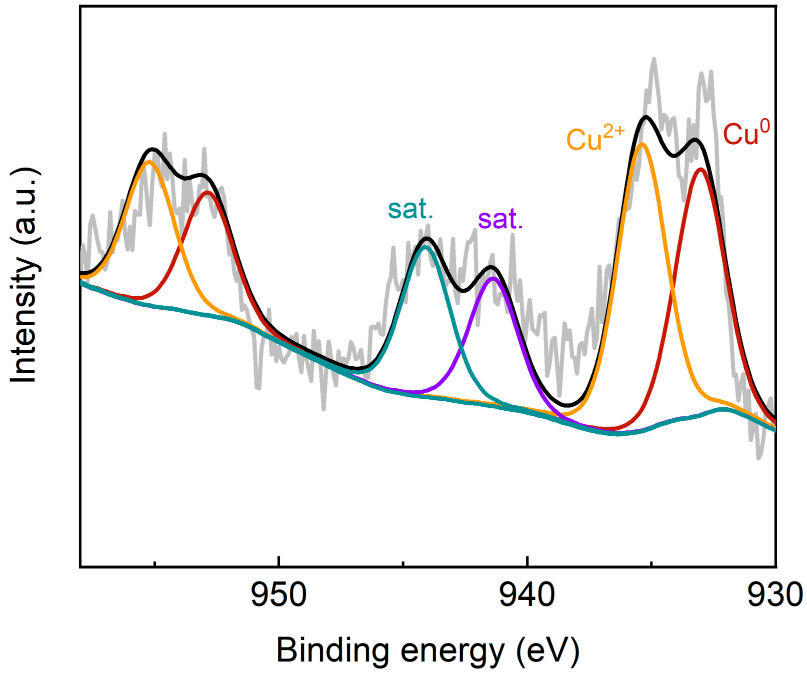

Figure S4. High-resolution XPS spectra of Cu 2p regions in RuCu-ANPs/IL-GA.

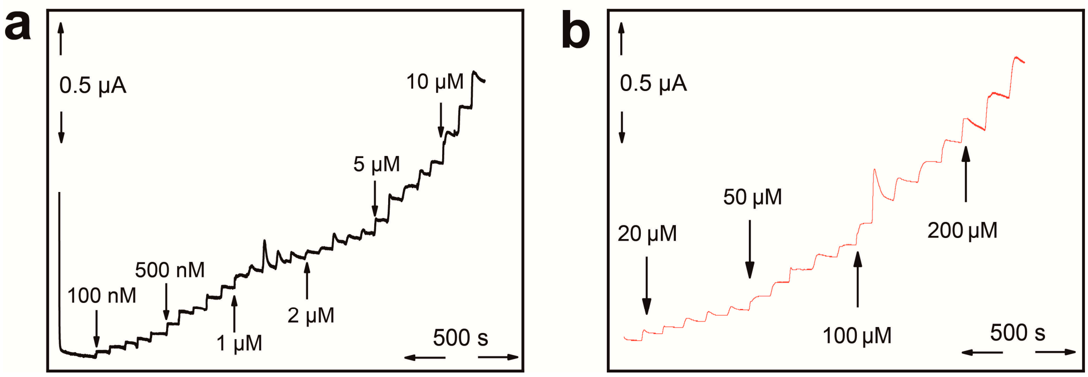

Figure S5. Amperometric current response of RuCu-ANPs/IL-GA based sensor to successive addition of low concentration (100 nM-10 μM) and medium concentration (20 μM-200 μM) NO in PBS at 0.74 V (*vs.* Ag/AgCl).

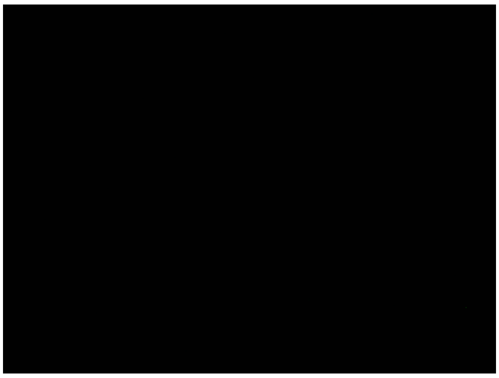

Figure S6. Dark-field fluorescent image of MCF-7 cells after the calcein-AM/PI assay to stain the viable cells green by calcein-AM and dead cells red by PI. The living cells were incubated with RuCu-ANPs/IL-GA for 72 h.

References

[1] Li J, Xie J, Gao L, Li CM. Au nanoparticles-3D graphene hydrogel nanocomposite to boost synergistically in situ detection sensitivity toward cell-released nitric oxide. ACS Appl Mater Interfaces. 2015,*7*(4):2726-2734. https://doi.org/10.1021/am5077777

[2] Li R, Qi H, Ma Y, Deng Y, Liu S, Jie Y, Jing J, He J, Zhang X, Wheatley L, Huang C, Sheng X, Zhang M, Yin L. A flexible and physically transient electrochemical sensor for real-time wireless nitric oxide monitoring. Nat Commun. 2020, 11(1):3207. https://doi.org/10.1038/s41467-020-17008-8
